# Sucralose: A Review of Environmental, Oxidative and Genomic Stress

**DOI:** 10.3390/nu17132199

**Published:** 2025-07-01

**Authors:** Volodymyr V. Tkach, Tetiana V. Morozova, Isabel O’Neill de Mascarenhas Gaivão, Natasha Gomes de Miranda, Yana G. Ivanushko, José Inácio Ferrão de Paiva Martins, Ana Novo Barros

**Affiliations:** 1 General and Material Chemistry Department, Chernivtsi National University, Kotrsyubynsky Str. 2, 58000 Chernivtsi, Ukraine; 2 Faculdade de Engenharia, Universidade do Porto, Rua Dr. Roberto Frias, s/n, 4200-065 Porto, Portugal; jipm@fe.up.pt; 3 Ecology and Environmental Protection Department, National Transport University, Omelianovych-Pavlenko Str. 1, 01001 Kyiv, Ukraine; tetiana.morozova@ukr.net; 4 Veterinary and Animal Research Centre (CECAV), University of Trás-os-Montes e Alto Douro (UTAD), 5000-801 Vila Real, Portugal; igaivao@utad.pt (I.O.d.M.G.); natashagomesdemiranda@gmail.com (N.G.d.M.); 5 Disaster and Military Medicine Department, Bukovinian State Medical University, Teatralna Sq. 9, 58001 Chernivtsi, Ukraine; yana_iv@ukr.net; 6 Centre for the Research and Technology of Agro-Environmental and Biological Sciences (CITAB), University of Trás-os-Montes e Alto Douro (UTAD), 5000-801 Vila Real, Portugal

**Keywords:** chloroorganic compounds, sucralose, oxidative stress, environmental persistence, genotoxicity, mutagenesis, aquatic toxicity, environmental safety assessment

## Abstract

This review explores current knowledge on the environmental, oxidative, and genomic effects of sucralose (E955), an artificial sweetener widely used in food products, including those for children, and known to cross both the placental barrier and into breast milk. Although initially considered safe, research conducted over the past two decades has presented conflicting evidence regarding its long-term impact, particularly on ecosystems and biological systems. Structurally similar to chlorinated compounds such as perfluoralkyl substances (PFAS), sucralose is highly persistent in the environment, which complicates its degradation and removal, especially from aquatic systems. Several studies have reported behavioral, metabolic, and even genomic alterations in aquatic organisms exposed to sucralose, raising concerns about its broader ecological safety. In addition, its presence has been linked to shifts in microbiota composition in both environmental and human contexts. Reports of sucralose-induced oxidative stress further highlight the need for caution in its continued use, particularly in sensitive formulations. Given its widespread presence and resistance to degradation, further investigation into the environmental and biological safety of sucralose is urgently needed.

## 1. Introduction

**Figure 1:**
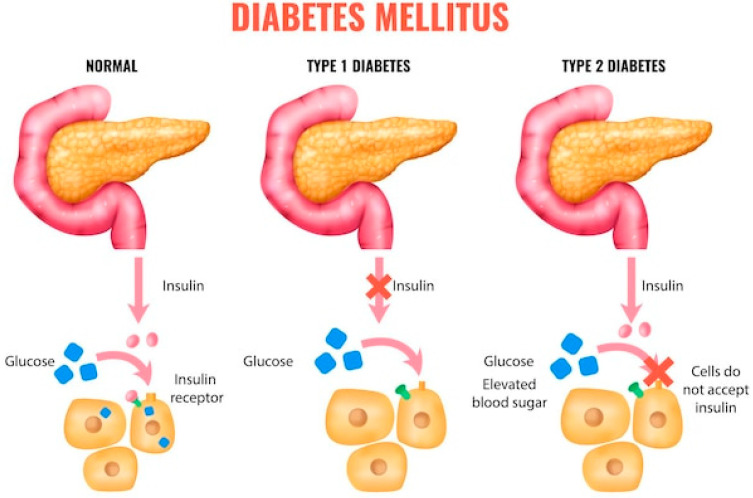
Diabetes mellitus etiology.

Diabetes mellitus (DM) type 1 and 2 [1,2,3] is a chronic metabolic disease characterized by impaired regulation of blood glucose levels due to either insulin deficiency or reduced insulin sensitivity. According to the World Health Organization, the prevalence of DM is rapidly increasing, affecting not only adults but also children and adolescents—a concerning trend for healthcare systems worldwide. In total, DM and cancer are responsible for nearly two thirds of the deaths registered worldwide (Figure 1):

Particular attention is given to specific forms of diabetes, including gestational, hereditary (monogenic), congenital, and juvenile diabetes. Gestational diabetes mellitus (GDM) develops during pregnancy and can lead to complications for both mother and fetus. Although it usually resolves after delivery, women with GDM are at significantly higher risk of developing type 2 diabetes later in life [4].

Both type 1 diabetes (T1D) and type 2 diabetes (T2D) have a genetic basis [4,5,6]. In T1D, key genes involved include *HLA-DR3* and *HLA-DR4*, located on chromosome 6 and responsible for immune regulation, as well as *INS* (directly responsible for insulin secretion), *PTPN22*, *IL2RA*, and *CTLA4*, which encode components of the immune response. In contrast, genetic predisposition in *T2D* is even stronger and involves at least eight genes: *TCF7L2*, *PPARG*, *KCNJ11*, *ABCC8*, *FTO*, *SLC30A8*, *HHEX*, and *CAPN10*. These genes regulate carbohydrate and fat metabolism, pancreatic function, and adipocyte formation and growth.

Hereditary forms of diabetes also include rare monogenic variants such as MODY (Maturity-Onset Diabetes of the Young), which follows an autosomal dominant inheritance pattern caused by specific mutations in genes such as *HNF4A*, *GCK*, *HNF1A*, *HNF1B*, *PDX1*, and *NEUROD1*. These forms typically present in young individuals without obesity or insulin resistance [5,6]. Differentiating MODY from classical type 1 or type 2 diabetes is diagnostically and therapeutically important, particularly when deciding on the need for insulin therapy.

Congenital or neonatal diabetes is usually diagnosed within the first six months of life and is associated with genetic mutations impairing insulin secretion. Although rare, early diagnosis is critical to enable personalized treatment strategies [7].

Juvenile diabetes, most commonly type 1 diabetes, results from autoimmune destruction of pancreatic β-cells. It primarily affects children and adolescents and requires lifelong insulin therapy and daily glucose monitoring. The incidence of type 1 diabetes in children has been rising over recent decades, likely due to a combination of genetic predisposition and environmental factors [8,9].

Given the variety of diabetes forms and their underlying mechanisms across age groups, studying its etiology, pathogenesis, clinical features, and diagnostic and therapeutic approaches remains highly relevant. An interdisciplinary approach is essential to improve patients’ quality of life and develop effective prevention programs. Moreover, the expression of the aforementioned genes is directly related to the human perception of sweet taste and has to be considered during the development of novel sweeteners.

In light of the global increase in diabetes prevalence, there is growing interest in dietary factors that may influence disease risk or progression. One key strategy in diabetes prevention and management is reducing sugar intake, which has led to the widespread adoption of artificial and high-intensity sweeteners.

Saccharin was the first synthetic sweetener synthetized in 1879 from toluene by Remsen and Fahlberg [10]. Alongside with dulcin, cyclamate and aspartame are the earliest sweeteners known. Nonetheless, dulcin hepatotoxicity has been confirmed unsafe and it was banned in the 1950–1960s. As for saccharin, cyclamate and aspartame, they are still in use. Nevertheless, their disadvantages, like bitter aftertaste and possible adverse effects led to the search of alternative and sucralose seemed to be a viable alternative.

**Figure 2:**
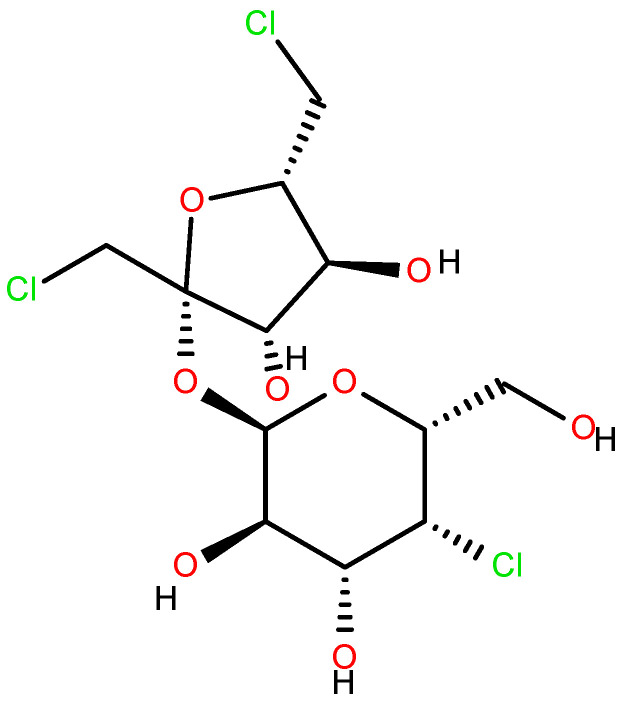
Sucralose.

Sucralose (Figure 2) [11,12,13,14,15] was first synthesized in 1976 by Tate & Lyle, along with other chlorinated carbohydrates. Its extremely sweet taste was discovered accidentally, due to a linguistic misunderstanding by one of the researchers who “tasted” rather than simply “tested” the compound. Since then, sucralose’s “sweet” journey began, and it remains one of the most widely used sweeteners worldwide [16,17,18].

Although sucralose is widely considered safe for general consumption, its potential health and environmental impacts have not been fully elucidated. Several of its adverse effects have only been identified within the past decade and are still under active investi-gation. Moreover, even considering that 85% of sucralose taken by mouth remains un-changed, the direct and indirect metabolic influence (including the metabolic paths in different normal and pathological conditions) of the other 15% is still pending thorough in-vestigation (See the review article [19] and the figures therein).

Recent studies involving pregnant and breastfeeding women [19,20,21,22] have confirmed the presence of sucralose in breast milk, where it may cause irreversible disruptions to the development of the fetal gut microbiota during late pregnancy, as well as in neonates and infants. This raises ongoing concerns regarding the safety of sucralose during pregnancy and lactation.

In addition, sucralose exhibits low biodegradability, which contributes to its persistence and accumulation in the environment [23,24]. Upon thermal degradation or microbial transformation, it can generate toxic by-products such as dioxins and tetrachlorodibenzofurans. Another area of concern is the oxidative stress that sucralose may induce in various organisms, along with the mechanisms for mitigating such effects [25,26]. Notably, the potential impact of sucralose on the oxidative or environmental stress responses of other substances remains insufficiently studied.

A further critical concern involves the detection of 6-acetyl sucralose—an industrial precursor to sucralose [27]—in appreciable concentrations within commercial product samples. Its formation in the human intestine is also plausible, as it can undergo esterification at the hydroxyl group attached to the C6 carbon atom. Recent studies have demonstrated the genotoxicity of 6-acetyl sucralose [27,28,29,30].

Toxicological analyses of this sterically modified derivative have indicated that its genotoxic effects are likely clastogenic, causing structural DNA damage. Even trace levels of 6-acetyl sucralose detected in processed food and beverages may exceed the recommended safety threshold of 0.15 μg/person/day. Moreover, this compound has been shown to upregulate gene expression in intestinal epithelial cells associated with inflammation, oxidative stress, and carcinogenic processes, including *MT1G* and *SHMT2*.

**Figure 3:**
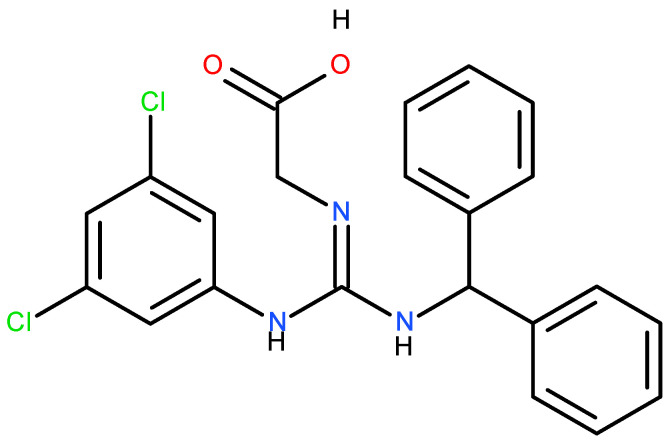
Carrelame, a chloroorganic supersweetener.

Another harmful action of the steric derivative impedes the action of CYP1A2 and CYP2C19, proteins of the cytochrome P450 family, responsible for the transformation of various food substances to their more accessible form, which leads to secondary toxic effects [19,27]. The increase in the genotoxicity of 6-acetyl sucralose concerning sucralose is due to the more significant activity of the secondary organic chloride, linked to the C4 carbon atom (Figure 2), activated by the accepting action of the steric group. Moreover, the effect of the presence of chlorine in sucralose is also important in the aspect of the possible food use, oxidative stress, genotoxicity and environmental fate of the supersweetener carrelame (Figure 3), which is also a chloroorganic compound.

The data concerning the environmental impact and bioaccumulation of sucralose is also controversial. For example, the 2008-dated report [31] by Swedish Environmental Institute indicates sucralose is a compound, which does not alter survival, growth and reproduction of aquatic organisms (plants, algae, crustaceans and fish) at concentrations 9000 times higher than those detected in the environment. Nevertheless, as the sucralose consumption growth constantly, especially during and after COVID-19 pandemics, considering its low biodegradability rate, the sucralose environmental concentration constantly grows and, if all remains unchanged, will reach and overcome the threshold, which is reinforced by a more recent, 2024-dated, investigation by the Swedish University of Agricultural Sciences [32]. Moreover, the sucralose environmental fate in the presence of other environmentally aggressive substances is still subject of an extensive study.

In recent years, dietary patterns in high-income countries—including EU Member States, the United Kingdom, the United States, Australia, New Zealand, Japan, and South Korea—have shifted toward health-oriented consumption, with growing attention to the role of bioactive compounds in promoting well-being. In the European context, the increasing interest in functional foods and beverages has been driven by consumer demand for nutritional benefits that extend beyond basic nourishment. As a result, the consumption of products rich in bioactive compounds, such as polyphenols, flavonoids, and vitamins, has steadily risen [32,33], largely due to their antioxidant properties and perceived health-promoting effects. Reports indicate that the European functional food market is undergoing stable expansion, sustained by concerns related to disease prevention and long-term health maintenance [34,35,36]. Within this context, the inclusion of antioxidant bioactive compounds in dietetic formulations may help attenuate oxidative stress associated with synthetic additives, such as sucralose.

Similarly, in the United States, there has been a measurable increase in the per capita intake of dietary antioxidants from plant-based sources. This trend reflects both enhanced awareness of the protective effects of bioactive compounds and their growing availability in consumer products. The scientific literature increasingly supports the role of polyphenols in mitigating oxidative damage, highlighting their relevance not only in health promotion but also as sustainable alternatives to synthetic ingredients like sucralose [37,38,39].

This review compiles and analyzes findings from the past 15 years on the environmental fate, oxidative stress potential, and genotoxic effects of sucralose, aiming to clarify its implications for both human health and ecological integrity. Special attention is given to its persistence in aquatic systems, its impact on cellular homeostasis, and the emerging strategies for sucralose recovery and recycling in the context of circular economy principles, all of which are critical considerations for achieving food safety and sustainable nutrition.

## 2. Materials and Methods

Articles for this review were selected using a structured and transparent methodology. A comprehensive literature search was conducted across major scientific databases, including Web of Science, Scopus, and PubMed, using targeted keywords related to sucralose’s environmental fate, ecotoxicity, genotoxicity, oxidative stress, and recycling.

Inclusion criteria focused on the relevance to the review topic, methodological rigor, and publication in peer-reviewed journals. To ensure a thorough and up-to-date overview of the field, additional references were identified through citation tracking. Following the initial screening of titles and abstracts, full-text articles were evaluated to confirm their relevance and quality.

This systematic approach ensured the inclusion of high-quality studies that provide valuable insights into the current advances and ongoing challenges in the detection and environmental assessment of sucralose.

## 3. Sucralose Environmental Stress Overview

### 3.1. Oxidative Stress of Sucralose

Oxidative stress is characterized by an imbalance between reactive oxygen species (ROS) production and the antioxidant defense mechanisms that counteract oxidative damage. When this balance is disturbed, cellular components such as lipids, proteins, and DNA may be compromised, with systemic consequences for tissue function and overall health.

Over the past 15 years, increasing attention has been given to the potential of artificial sweeteners, particularly sucralose, to induce oxidative stress. Numerous studies in both in vitro and in vivo models have documented varying degrees of ROS accumulation and its consequences in different biological systems [40,41,42,43,44,45,46,47,48,49,50,51,52,53,54,55,56,57,58,59,60,61,62,63,64,65,66,67,68,69,70].

#### 3.1.1. Cellular and Molecular Effects

In mesenchymal stromal cells, sucralose exposure significantly increased ROS levels after three days, triggering an acute inflammatory response and stimulating adipogenic differentiation [45]. These findings suggest a dose-dependent mechanism through which sucralose may contribute to metabolic dysregulation and heightened cardiovascular risk.

Similarly, studies in zebrafish (*Danio rerio*) embryos revealed that even environmentally relevant concentrations of sucralose elevate ROS production and promote the overexpression of pro-apoptotic and oxidative stress–related genes such as *Nrf1*, *Nrf23*, *CASP3*, and *CASP9*. These molecular alterations were associated with embryonic malformations and apoptosis [46].

In human microglial cells, Hacioglu [47] demonstrated that chronic sucralose exposure interferes with neuroimmune regulation through pathways involving SIRT/NLRP3/IL-1β/GPx4. The treatment reduced cell viability, increased caspase activity, and disrupted antioxidant balance, raising concerns about sucralose’s potential role in neuroinflammation and neurodegenerative processes.

#### 3.1.2. Systemic and Organ-Specific Toxicity

The systemic implications of sucralose-induced oxidative stress are further supported by studies in mammals. Hu et al. [50] found that sucralose enhances the toxicity of benzo(a)pyrene in mice by inhibiting PGP-mediated efflux, leading to compound accumulation, increased ROS, and greater cytotoxicity. Additionally, alterations in renal tissue structure were observed.

In the gastrointestinal tract, high doses of sucralose compromised epithelial barrier integrity by reducing claudin-3 levels and increasing permeability, with potential implications for gut inflammation and systemic endotoxemia [51]. Elveren [52], studying *Triticum aestivum*, also identified oxidative stress markers such as increased peroxidase activity, confirming cytotoxic effects across plant and animal models.

Liver and cardiovascular tissues appear particularly susceptible. Wu et al. [58] reported that sucralose exacerbates non-alcoholic fatty liver disease (NAFLD) in mice via T1R3 receptor activation, which stimulates ROS production. Basson [56] found sucralose to upregulate PPAR-α, affecting lipid metabolism and energy homeostasis. Similarly, long-term consumption of FDA-approved sweeteners, including sucralose, led to lipid imbalances and structural changes in cardiac tissue [55].

Hepatotoxicity was also observed by Haq et al. [57], who described morphological alterations in hepatocytes, with aspartame showing even greater toxicity. El-Haddad [65] confirmed similar hepatic and renal inflammatory responses, pointing to sucralose’s pro-oxidant effects during chronic exposure.

#### 3.1.3. Interactions with Gut Microbiota and Immune System

Sucralose’s impact on gut microbiota and immune modulation has emerged as another important research focus. Jarmakiewicz-Czaja et al. [64] showed that sucralose, particularly in Western-style diets, contributes to microbial dysbiosis and liver metabolic dysfunction, driven by oxidative and inflammatory responses. El-Tahan et al. [67] observed that sucralose may both promote beneficial bacterial species and disrupt metabolic signaling, depending on dosage and exposure duration.

Yu et al. [62] highlighted a concerning mechanism by which sucralose promotes horizontal gene transfer of antibiotic resistance genes (ARGs) via ROS-mediated conjugation in bacteria. This suggests sucralose may indirectly contribute to antimicrobial resistance.

#### 3.1.4. Neurotoxicity and Behavioral Outcomes

The neurotoxic effects of sucralose have also been documented in aquatic organisms. In *Daphnia magna*, combined exposure to sucralose and acesulfame resulted in dose-dependent behavioral and cardiac changes, as well as altered acetylcholinesterase activity [69]. In *Cyprinus carpio*, chronic exposure led to DNA damage, increased ROS, and apoptosis in blood cells [66].

Zhang et al. [63] found that while moderate sucralose doses extended lifespan in Caenorhabditis elegans, higher concentrations induced oxidative damage and reduced longevity. In humans, increased sucralose intake has been associated with neurovascular and cognitive changes, especially in diabetic and obese individuals, raising concerns about its role in neurodegenerative disease onset [70].

#### 3.1.5. Environmental and Thermal Degradation

Another emerging concern is the environmental and thermal degradation of sucralose. Eisenreich et al. [53] found that heating sucralose-containing products can lead to the formation of dioxins and other chlorinated organic compounds, which significantly enhance oxidative stress. Zhai et al. [68] further showed that under UV exposure, sucralose degrades into mutagenic chloroorganics capable of damaging bacterial DNA.

#### 3.1.6. Dietary Context and Mitigation Strategies

Importantly, several studies emphasize that sucralose’s effects are highly context-dependent. Bórquez et al. [59] observed changes in mitochondrial bioenergetics without ATP depletion in intestinal cells. Meanwhile, Heredia-García et al. [60] and Saad [61] stressed the synergistic effects of sucralose when combined with other sweeteners or consumed regularly. Elveren [52] and Singh et al. [55] suggest that polyphenol-rich diets may counterbalance these effects. Similarly, stevia demonstrated anti-inflammatory potential compared to sucralose and sucrose [54].

In summary, the available literature consistently links sucralose exposure—particularly at high or prolonged doses—to oxidative stress and a range of downstream physiological disturbances, from inflammation and apoptosis to neurotoxicity and metabolic dysregulation. These findings, summarized in Table 1, highlight the need for more comprehensive risk assessments and long-term studies addressing chronic exposure, vulnerable populations, and combined dietary effects.

Therefore, sucralose does cause oxidative stress in different organisms. Moreover, it enhances the oxidative stress of other similar and not similar substances by facilitating their penetration through the cell membrane. Also, the sucralose-induced oxidative stress may result in genotoxicity in different organisms. The genomic stress, caused by sucralose will be described in detail in Section 3.2.

### 3.2. Genomic Stress Induced by Sucralose

As a chlorinated organic compound, sucralose belongs to a chemical class that includes substances with well-documented genotoxic properties. Consequently, its potential to induce genotoxic effects has been widely investigated over the past decades [62,66,68,71,72,73,74,75,76,77,78,79,80,81,82,83,84,85]. However, the outcomes of these studies, including those examining sucralose’s interactions with DNA and its ability to trigger oxidative stress leading to DNA damage, have been markedly inconsistent. While early reports suggested a lack of genotoxicity [71,72], more recent research—some of which has already been discussed in the previous section [66,68]—has provided compelling evidence of genotoxic effects associated with sucralose exposure.

In a 2010 study [71], genotoxicity was assessed using four different assays: the Ames reverse mutation test in *S. typhimurium*, the *E. coli* pol A+/A– test, an in vitro chromosomal aberration test in human lymphocytes, and a mutation test in TK+/− mouse lymphoma cells. Additionally, in vivo chromosome aberration and micronucleus tests were performed in rats and mice. Despite the comprehensive nature of the testing battery, the study did not explore other genotoxicity assays such as SMART, TUNEL, or Comet—used in subsequent studies [66]—nor did it examine long-term or high-dose exposures, or possible synergistic effects from co-exposure with other sweeteners. Based on the available data, the authors concluded, perhaps prematurely, that sucralose does not present genotoxic risks.

Similarly, a comparative evaluation of several sweeteners—sucralose, acesulfame K, aspartame, saccharin, and steviol glycosides—by [72] largely affirmed the absence of genotoxicity for sucralose. However, a closer reading of the referenced data within the study reveals inconsistencies that leave room for alternative interpretations. Notably, there remains a lack of data assessing genotoxic effects when sucralose is combined with other carbohydrates or sweeteners.

Other studies have raised more serious concerns. For instance, ref. [73] reported that thermal degradation products of sucralose are toxic and capable of reacting with DNA. This study, which evaluated sucralose’s cyto-, geno-, and immunotoxicity through in vitro and in silico methods, found a non-selective reduction in CD4+ and CD8+ lymphocyte populations, along with dose-dependent DNA alterations and chromosomal structural changes in lymphocytes. These effects were associated with the modulation of genes such as *MAPK8*, *APTX*, and *EID1.*

An in silico study [74] investigated the mutagenic and carcinogenic potential of 16 artificial sweeteners using predictive tools such as LAZAR, pKCSM, and Toxtree. The results identified sucralose, along with glucin, 5-nitro-2-propoxyaniline, and acesulfame K, as potentially mutagenic. Glucin and 5-nitro-2-propoxyaniline were also predicted to be carcinogenic.

Studies on *Allium cepa* provided further evidence of sucralose’s genotoxic potential. In [75], exposure to aspartame, sorbitol, and sucralose resulted in various chromosomal abnormalities, most notably micronuclei formation during interphase and mitosis in root tip cells—a key indicator of mutagenesis. A follow-up study [76] that examined sucralose, aspartame, and their combination revealed synergistic genotoxic effects in the same plant model.

Animal studies have also supported these findings. One of the earliest detailed investigations [77] exposed male Swiss mice prenatally to sucralose, observing a dose-dependent increase in hematopoietic neoplasms when sucralose was administered along with its hydrolysis product 6-CF at concentrations of 2000 ppm and 16,000 ppm. These findings suggest sucralose may influence epithelial and glandular tissues in both benign and malignant directions.

A study focused on e-liquids [78] examined the thermal degradation of sucralose when used as a sweetener in vaping products. The authors highlighted that several sucralose dehydration products are either carcinogenic or genotoxic. The inhalation route of exposure may present different biological impacts compared to ingestion, raising concerns about potential synergistic genotoxicity between sucralose and its degradation products.

Van Eyk [79] tested several sweeteners, including sucralose, on Caco-2, HT-29, and HEK-293 cell lines. Among all sweeteners evaluated, saccharin and sucralose caused the greatest degree of DNA fragmentation across all tested cell types.

In hepatic cell cultures, Dhurandhar et al. [80] observed genomic alterations and increased reactive oxygen species (ROS) levels in liver tissues from albino rats administered sucralose. These findings led the authors to caution against unregulated sucralose consumption due to potential irreversible liver damage.

The study by [81] evaluated the influence of three sweeteners—acesulfame K, sucralose, and aspartame—on *E. coli*. While acesulfame K enhanced bacterial growth and aspartame had a highly inhibitory effect, sucralose moderately influenced gene expression. Notably, sucralose exposure upregulated genes such as *adk*, *fabI*, and *lpd*, which are involved in vitamin and fatty acid metabolism, the citrate cycle, and amino acid degradation. Although sucralose had the least pronounced effect among the three, the study did not investigate possible synergistic effects when sweeteners are used in combination.

In a clinical context, [82] examined inflammatory transcriptomic responses in overweight and obese women following consumption of diet sodas containing sucralose and acesulfame-K. A total of 828 genes were significantly upregulated post-ingestion, including *IL-6*, *IL-1α*, *IL-1β*, *TNF*, and *NF-κB*—genes linked to lipid metabolism and metabolic regulation. These results are consistent with findings in mice reported by Olivier-Van Stichelen et al. [83], who showed alterations during pregnancy and lactation. A subsequent study [84] by the same research group demonstrated that sucralose and acesulfame-K modulate P-glycoprotein (PGP) expression in the rat colon, acting as inhibitors and affecting substrate transport.

The condensed review of the data about sucralose genomic stress may be represented in Table 2:

As the current literature shows, there is still no consensus regarding the genotoxic potential of sucralose or the precise mechanisms underlying its effects. However, more recent studies published between 2022 and 2025 increasingly suggest a possible capacity for sucralose to induce DNA alterations. Notably, its genotoxicity in plants remains underexplored, despite the relevance of this area due to the structural similarities between sucralose and various herbicides. In addition, studies investigating the genotoxicity of sucralose in combination with other sweeteners, carbohydrates, or dietary components, as well as in the presence of food contact materials such as bisphenols—which may migrate into food from packaging—are still lacking and warrant further investigation.

Furthermore, the presence of sucralose precursors such as 6-acetylsucralose [27,28,29,30,85,86], along with its hydrolysis product 6-chlorofructose (6CF), may exacerbate the genotoxic potential of sucralose and its mixtures with both synthetic and natural food components. Thus, clarifying the genotoxicity of sucralose—both alone and in combination with other compounds—remains a critical issue that requires comprehensive and multidisciplinary research. This is also essential for evaluating the environmental persistence and ecological impact of sucralose, its potential to disrupt ecosystems and biological homeostasis, and for developing strategies to mitigate or recycle its excess concentrations. Such efforts are aligned with the principles of the circular economy and the goals of the Fourth Industrial Revolution.

### 3.3. Environmental Stress of Sucralose

**Figure 4:**
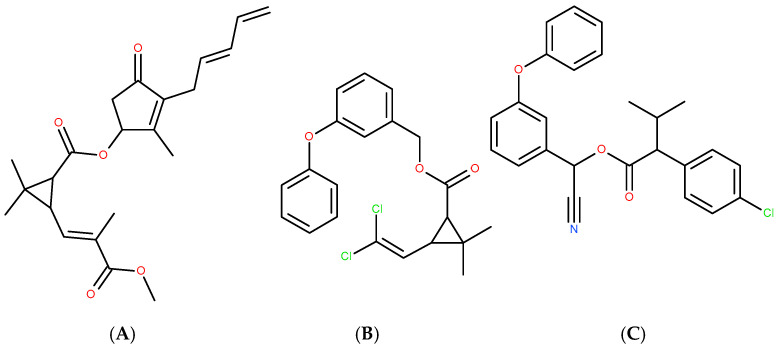
Natural pyrethrin (**A**), and synthetic chlorine-introduced permethrin (**B**) and fenvalerate (**C**).

Chloroorganic compounds constitute one of the most important and contradictory classes of organic compounds of both natural and synthetic origin [16,87,88,89,90,91,92,93]. Natural chloroorganic compounds include antioxidants (chlorinated polyphenols, steroids, aminoacids) and alkaloids, which modulate and regulate either oxidative or environmental stress. They also play the role of natural pesticides. Some of the synthetic chloroorganic compounds mimic the properties of natural organochlorines, which may serve as a strategy for the synthesis of naturally inspired drugs. In some cases (permethrin and fenvalerate in relation of the parent natural compound pyrethrin [93]—Figure 1), chlorine atoms are introduced into an initially chlorine-free molecule (Figure 4) in order to enhance insecticide, acaricide and arachnicide properties. Nevertheless, the realization of this strategy has to be realized with caution and following a detailed approach [94,95,96], due to the environmental effects of these compounds.

Sucralose (Figure 2) [97,98,99,100,101] is another example of chlorine being introduced into a molecule that originally lacked chlorine (sucrose and semisynthetic galactosaccharose). As an organochlorine compound (chlorohydrin), sucralose is not metabolized by most living organisms. Consequently, it accumulates in the environment, and its concentration continues to rise. This underlines the urgency of investigating its environmental impact, including stress induction, environmental fate, removal, and reuse.

The assessment of sucralose’s industrial production and environmental behavior has gained increasing relevance, particularly in light of its emerging role as a tracer of anthropogenic activity and pollution sources [102,103,104,105,106,107,108,109,110,111,112,113,114,115,116,117,118,119,120,121,122,123,124,125,126]. A pioneering study [102] conducted the first cradle-to-factory-gate life cycle assessment (LCA) of sucralose production from cane-derived sucrose in the United States, estimating a global warming potential of 71.82 kg CO_2_-eq per kilogram of sucralose produced. This high carbon footprint was largely attributed to the use of highly ecotoxic reagents during the synthetic process. The research was subsequently expanded within the framework of the SWEET project [103].

Environmental monitoring studies have further confirmed sucralose’s persistence. Young et al. [104] detected concentrations of 26 ± 2 ppb in surface waters and 20 ± 6 ppb in outfall samples from wetlands. Their findings revealed that sucralose undergoes neither significant adsorption nor chemical transformation in such environments, underscoring its environmental stability. Similarly, ref. [105] predicted sucralose concentrations in reclaimed water scenarios to be on par with those of per- and polyfluoroalkyl substances (PFAS), raising the possibility of classifying sucralose as a persistent halogenated organic compound. The structural resemblance between sucralose and PFAS also suggests the potential applicability of PFAS removal strategies for the adsorption, removal, and recovery of sucralose.

In addition, sucralose has been proposed as an anthropogenic marker. In [106], both sucralose and caffeine were detected in coral reef ecosystems, indicating their bioaccumulation within the food web of Vatia Bay (American Samoa) as a result of domestic wastewater discharge. Likewise, ref. [107] identified these compounds as reliable indicators of household wastewater contamination in the Laurentian Great Lakes basin.

The ecological impact of sucralose on microbial communities has also been explored. In [108], exposure to sucralose resulted in a dose-dependent reduction in microbial and diatom activity, alongside an increase in cyanobacterial abundance in marsh ecosystems—suggesting a shift in community composition. Among the four sweeteners tested (acesulfame K, cyclamate, saccharin, and sucralose), sucralose exhibited the highest persistence, with negligible degradation observed during conventional water treatment. While its acute ecotoxicity appears limited, its long-term ecological effects remain insufficiently characterized.

According to [109], sucralose has been consistently detected in municipal water supplies across both the EU and the US, with mean concentrations of approximately 10 μg/L. Although not classified as ecotoxic or bioaccumulative as of 2012, the need for continued investigation into its ecological safety has been recognized.

Further concerns arise from the thermal degradation of sucralose. In [110], FTIR and HRMS analyses revealed the formation of toxic polychlorinated aromatic compounds (polychloroarenes) upon dehydration of isolated sucralose, along with the release of CO_2_ and HCl. These findings raise important questions regarding the safety of sucralose when subjected to high temperatures, such as during baking or cooking.

Photodegradation studies, such as the one conducted in [111], demonstrated that sucralose can be nearly completely mineralized via a TiO_2_-catalyzed photo-Fenton reaction within five hours, as monitored by HPLC. However, other Splenda^®^ constituents—namely maltodextrin and dextrose—were only partially degraded. Although the Fenton process is effective, it may pose environmental risks due to the release of organically bound chlorine species. The fate of this chlorine, which can be oxidized into gaseous chlorine or reactive chlorine oxides (e.g., ClO_2_), was not fully addressed. These byproducts may present short-term environmental hazards that outweigh the risks posed by sucralose itself. For this reason, alternative remediation strategies such as cathodic reduction have been proposed.

**Figure 5:**
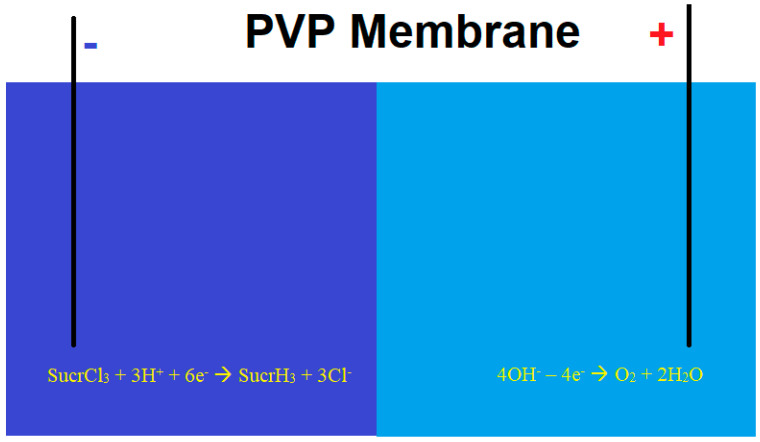
Sucralose cathodic dehalogenation using membrane electrolysis.

In [112,113], a sucralose removal process was proposed and theoretically modeled using a membrane-based electrolytic system involving cathodic dehalogenation, optionally assisted by bi- and trivalent vanadium compounds (Figure 5). To prevent chlorine evolution, the cathodic and anodic compartments are separated by a polyvynilpyridine (PVP)-based membrane, which restricts the migration of chloride ions to the anodic side. The anodic electrolyte is devoid of common anions, allowing for water electrolysis and oxygen evolution instead of chlorine gas production. A similar process has been employed to quantify other chlorinated organic compounds [114,115] through both direct and indirect approaches.

The work [116] was dedicated to the environmental fate of fourteen wastewater micropollutants, including sucralose, on lettuce and root-associated bacteria. Sucralose was among the three of the most concentrated pollutants in both leaves and roots of lettuce. Moreover, its presence highly affected the root microbiota, possible due to the detoxication enzyme inhibition and absorption rate enhancement.

In [117], biochar from recovered cellulose was used for sucralose removal from the environment. This approach is important not only from the point of view of wastewater reuse, but also from the point of view of sucralose removal and reuse as secondary raw material with aim to convert it into more environmentally secure substances. It was shown that the recovered cellulose had excellent potential for sucralose recovery, contrarily to NSAID found in the environment.

Wu et al. [118] developed an ultra-high-performance liquid chromatography tandem mass spectrometry (UHPLC–TMS) method capable of detecting nanomolar concentrations of sucralose and acesulfame K in groundwater samples collected from domestic wells in Alberta, Canada. The method achieved detection limits as low as 200 pg/L for acesulfame K and 5 ng/L for sucralose. Frequent detection of both sweeteners in private wells highlights the importance of continuous monitoring to ensure water quality and public health safety.

A comprehensive assessment of sucralose and acesulfame K as emerging water contaminants was presented in [119], supported by LC-MS analysis of both surface and groundwater samples from the Danube and Sava Rivers near Belgrade, Serbia. The study revealed widespread contamination, with sucralose concentrations reaching up to 4756 ng/L. Although these concentrations were not found to pose immediate acute risks to aquatic organisms, the authors emphasized the potential for seasonal fluctuation—particularly during the summer months, when increased human activity may contribute to elevated contaminant levels. Given sucralose’s low biodegradability, its persistence and bioaccumulation in freshwater systems—and potentially in downstream marine environments such as the Black Sea—remain areas of significant environmental concern.

In [32], the trends in sweetener consumption before, during, and after the COVID-19 pandemic were evaluated in Sweden through wastewater analysis, applying a wastewater-based epidemiology approach. No significant increase in sweetener concentrations was observed during the pandemic. Sucralose concentrations in wastewater ranged between 0.5% and 2% of the upper thresholds established by the European Union and the United Nations.

Overall, research trends indicate that sucralose tends to accumulate in aquatic and soil ecosystems, acting as a persistent environmental pollutant. However, its current concentrations are generally below levels that would cause significant ecological harm. Nonetheless, the need to monitor, quantify, remove, and potentially reuse sucralose in the environment is becoming increasingly important. Furthermore, a more comprehensive understanding of its environmental impact is required. In parallel, the substitution of sucralose with natural, non-carbohydrate-based sweeteners should also be considered to promote sustainable food production.

## 4. Discussion

### 4.1. Oxidative Stress Induced by Sucralose and Its Alleviation

As previously discussed, sucralose has been shown to induce oxidative stress [53,60,61,62,63,64,65,66,67,68,69,70,120]. The mechanisms involved may vary depending on the organism and the specific tissues affected, as well as the presence of other compounds. Notably, the oxidative stress caused by sucralose may be influenced or exacerbated by co-exposure to other substances, and vice versa. This is particularly relevant given the known synergistic effects between sucralose and other compounds in inducing oxidative stress.

In this context, the study by [121] and related works describe the behavioral and physiological effects of bisphenol A (BPA) in Drosophila melanogaster, largely attributed to BPA-induced oxidative stress. Furthermore, ref. [122] reports a type 2 diabetes-like condition in Drosophila, aggravated by a high-sucrose diet, which led to fibrosis and dysfunction in the Malpighian tubules. Similar enhancements of oxidative stress have also been observed with other bisphenols [123,124]. These findings raise the possibility that sucralose, commonly co-present with BPA in packaged foods, could amplify BPA-induced oxidative stress—just as it has been observed to intensify the effects of benzo[a]pyrene [50].

On the other hand, certain polyphenolic compounds—such as luteolin [125,126] and cerium oxide nanoparticles [127]—have demonstrated protective effects against BPA-induced oxidative stress. This suggests that the same or similar compounds could potentially mitigate oxidative stress induced by sucralose. Consequently, the incorporation of polyphenols in sucralose-containing dietetic beverages may help reduce oxidative damage, provided the antioxidant dosage is properly calibrated. This opens the door to the rational formulation of sucralose-based beverages enriched with polyphenols. Additionally, some polyphenolic compounds could serve as natural sweeteners, potentially replacing sucralose—a possibility further explored in Section 4.3.

Moreover, since sucralose and sugar are frequently combined in dietetic beverages, it is essential to better understand their combined oxidative and genotoxic potential.

### 4.2. State on Genomic Stress Induced by Sucralose

The genotoxicity of sucralose remains poorly understood, and a clearer understanding is essential to safeguard genomic integrity and homeostasis. While animal studies on sucralose genotoxicity report conflicting results, its potential genotoxic effects in plants are largely unexplored. This is particularly concerning given that sucralose is structurally related to organochlorine compounds found in pesticides and chemical warfare agents—compounds with well-documented genotoxic potential [128,129,130,131].

Under natural conditions, detecting sucralose-induced plant genotoxicity is challenging. Hence, in vitro and in vivo studies using plant cells are needed to determine whether sucralose can induce genetic mutations or other alterations. Given current data suggesting both direct and indirect genomic interactions in various organisms, further studies are necessary to investigate the genotoxicity of sucralose, particularly in combination with other food additives. This is especially relevant in the broader context of genotoxicity associated with artificial sweeteners [132,133,134].

### 4.3. Environmental Stress and the Need for Sucralose Substitution

The environmental stress associated with sucralose is increasing due to its exceptional persistence—comparable to that of fluorinated organic compounds. If current trends continue, environmental concentrations of sucralose may eventually surpass critical thresholds, leading to pronounced oxidative and genomic effects in ecosystems. For this reason, it is urgent to develop methods for the safe removal, reuse, and substitution of sucralose [135,136,137,138,139,140].

Although Fenton-based processes have proven effective for sucralose degradation [135,136,137,138], they may not be environmentally viable due to the formation of ecotoxic inorganic chlorine byproducts. Additionally, sucralose degradation and dehydration can produce even more toxic intermediates. Therefore, safer and more sustainable removal and reuse strategies are urgently needed.

In this context, several natural sweeteners have been proposed as “green” alternatives to sucralose [141,142,143,144,145,146,147]. These compounds are generally more bioavailable and biodegradable, and some even exhibit a higher sweetness index than sucralose. Their respective advantages and disadvantages are summarized in Table 3.

All of the sweeteners listed are naturally derived and non-toxic, and several—such as perillartine and neohesperidin—exhibit sweetness levels three to five times greater than that of sucralose (perillartine is already in use, but exclusively in Japan). In addition to their sweetening capacity, some of these compounds possess antioxidant properties, which makes them suitable for inclusion in sucralose-based beverages to potentially mitigate oxidative stress. Consequently, the substitution of sucralose with natural sweeteners, or their combined use in food formulations, emerges as both a feasible and necessary strategy for the development of environmentally safer dietetic products.

## 5. Conclusions

This comprehensive review highlights the multifaceted risks associated with sucralose, particularly its capacity to induce oxidative and genomic stress across various biological systems. The generation and accumulation of reactive oxygen species (ROS) triggered by sucralose exposure underpin its pro-oxidant effects, which lead to alterations in cellular metabolism, cytotoxicity, and DNA damage. These effects are further complicated by sucralose’s ability to enhance the toxicity of other chemicals, either through synergistic interactions or by modulating their metabolism and impairing cellular detoxification processes. Such mechanisms contribute to the disruption of gut microbiota homeostasis, which is increasingly recognized as a critical factor in human health.

Despite numerous studies, significant gaps remain in understanding the combined effects of sucralose with other dietary or environmental contaminants, including common food additives, bisphenols, and compounds found in electronic cigarette aerosols. The potential for synergistic or cumulative toxicity in these contexts demands urgent and systematic investigation. Furthermore, sucralose’s role in facilitating horizontal gene transfer among bacteria raises concerns about its indirect contribution to the global challenge of antibiotic resistance.

On the genomic front, evidence of DNA alterations, chromosomal aberrations, and micronuclei formation—particularly in marine organisms and plant cells—underscores the genotoxic potential of sucralose. Considering sucralose’s chemical nature as a chlorinated organohalogen, its structural similarity to certain pesticides and chemical warfare agents known for genotoxicity cannot be overlooked. Thermal degradation studies revealing the formation of highly toxic byproducts, such as dioxins and polychlorinated aromatic compounds, further emphasize the need for caution, especially regarding food processing and thermal exposure.

From an environmental perspective, the persistence of sucralose in aquatic and terrestrial ecosystems presents an ongoing ecological challenge. Its resistance to biodegradation leads to accumulation in water bodies and soils, with potential long-term impacts on microbial communities and ecosystem function. Although current environmental concentrations have not yet been linked to pronounced ecological harm, the trajectory suggests escalating risks if mitigation measures are not implemented. This situation underscores the necessity for robust removal and recycling technologies aligned with sustainable development principles, such as the circular economy and the Fourth Industrial Revolution’s focus on resource efficiency and pollution reduction.

Traditional treatment methods like Fenton oxidation, while effective at degrading sucralose, pose secondary risks due to the formation of ecotoxic chlorine species. Therefore, emerging approaches, including cathodic dehalogenation and membrane technologies, warrant further development and optimization to ensure environmentally benign removal while enabling recovery of valuable byproducts.

In addressing the health and environmental concerns posed by sucralose, the incorporation or substitution with natural sweeteners emerges as a promising strategy. Many natural sweeteners not only match or exceed sucralose’s sweetness but also possess antioxidant properties that may counteract oxidative stress. Their biodegradability and lower ecological footprint further reinforce their suitability as sustainable alternatives in food and beverage formulations.

Looking forward, multidisciplinary research efforts are essential to fully elucidate the mechanistic pathways of sucralose-induced oxidative and genomic stress, its interactive effects with other contaminants, and its broader ecological impacts. Regulatory frameworks should also adapt to incorporate emerging evidence, guiding safe levels of sucralose use and encouraging innovation in green sweetener alternatives.

Ultimately, balancing consumer demand for low-calorie sweeteners with health and environmental safety requires a comprehensive understanding of compounds like sucralose, combined with proactive strategies for monitoring, mitigation, and substitution. This integrated approach will contribute to more sustainable food systems and healthier ecosystems in the long term.

## Figures and Tables

**Table 1:** Sucralose-induced oxidative stress effects observed across different experimental models (herein ↑ stands for activity growth, ↓ for decrease, → for resulting in).

Experimental Model	Dose/Concentration	Observed Effects	Reference
Mesenchymal stromal cells	1–5 mM	↑ ROS, acute inflammation, adipogenic differentiation	[45]
*Danio rerio* embryos	0.5–5 mg/L	↑ ROS, malformations, ↑ *Nrf1*, *CASP3*, apoptosis	[46]
Human microglial HMC3 cells	0.5–10 mM	↓ viability, ↑ caspase-3, oxidative imbalance via SIRT/NLRP3/GPx4 pathway	[47]
Mice exposed to benzo(a)pyrene	0.5 mg/kg	↑ renal toxicity, PGP inhibition, ↑ ROS	[50]
Intestinal epithelial Caco-2 cells	1–5 mM	↓ Claudin-3, ↑ permeability, apoptosis at high doses	[51]
*Triticum aestivum* (plant)	50–200 ppm	↑ peroxidase activity, ↓ growth, oxidative damage	[52]
Mice with NAFLD	1.5–5% in diet	↑ T1R3, ↑ ROS, exacerbated hepatic steatosis	[58]
Mouse liver and heart	5 mg/kg/day for 12 weeks	↑ PPAR-α expression, structural alterations, lipid imbalance	[55]
Mouse hepatocytes	15–60 mg/kg	Histological damage, inflammation, oxidative stress	[57,65]
Gut microbiota in rats	0.1–1% in diet	Dysbiosis, ↑ inflammation, hepatic metabolic changes	[64]
Gut bacteria (in vitro)	0.01–1 mM	↑ Horizontal gene transfer via ROS-induced SOS response	[62]
*Daphnia magna* (crustacean)	0.1–2 mg/L	Behavioral, cardiac, and AChE changes	[69]
*Cyprinus carpio* (fish)	5–20 mg/L for 21 days	↑ ROS, DNA damage, apoptosis in erythrocytes	[66]
*C. elegans* (nematode)	0.1–1% in diet	↑ lifespan at low doses; ↓ lifespan and ↑ oxidative stress at high doses	[63]
Heated sucralose-containing foods	120–180 °C	Formation of dioxins and chlorinated byproducts → ↑ oxidative stress	[53]
Sucralose + UV (aquatic exposure)	0.1–10 mg/L + UV	Genotoxic chlorinated byproducts, bacterial DNA damage	[68]
Humans (epidemiological data)	Dietary consumption	Association with neurovascular changes and inflammation in diabetic/obese individuals	[70]
Rats with polyphenol-rich diet	Sucralose + polyphenols	Polyphenols mitigate sucralose-induced oxidative stress and inflammation	[52,54,55]
Stevia comparison	Equivalent dietary doses	Stevia showed superior anti-inflammatory profile	[54]

**Table 2:** Sucralose-induced genomic stress and its manifestation.

Model/System	Type of Analysis	Key Findings	Reference
*S. typhimurium*, *E. coli*, human lymphocytes, mouse lymphoma cells, rodents	Ames, chromosomal aberrations, micronucleus test	Concluded absence of genotoxicity, though without long-term/high-dose or co-exposure analyses.	[71]
Multiple sweeteners (including sucralose)	Various genotoxicity tests	Mostly negative results for sucralose, but some referenced data suggest inconsistencies.	[72]
Human lymphocytes (in vitro), in silico	Cyto-, geno-, immunotoxicity; gene expression	Dose-dependent DNA/chromosomal damage; modulation of *MAPK8*, *APTX*, *EID1*.	[73]
In silico (LAZAR, pKCSM, Toxtree)	Mutagenicity and carcinogenicity prediction	Sucralose predicted to be mutagenic; glucin and 5-nitro-2-propoxyaniline carcinogenic.	[74]
*Allium cepa*	Chromosomal abnormalities	Induction of micronuclei and mitotic abnormalities by sucralose.	[75]
*Allium cepa*	Genotoxicity (single and combined exposure)	Confirmed synergistic genotoxicity of sucralose and aspartame.	[76]
Male Swiss mice (prenatal to adulthood)	Long-term exposure, tumor incidence	Increased hematopoietic neoplasms at high doses of sucralose and 6-CF.	[77]
E-liquids with sucralose	Thermal degradation analysis	Sucralose degradation products identified as carcinogenic/genotoxic.	[78]
Caco-2, HT-29, HEK-293 cells	DNA fragmentation	Sucralose showed highest DNA damage along with saccharin.	[79]
Hepatic cells from albino rats	ROS levels, genomic analysis	Genomic and oxidative stress induced in liver tissue.	[80]
*E. coli*	Transcriptomics, metabolic pathways	Sucralose modulated expression of metabolic genes; less disruptive than aspartame.	[81]
Human (overweight/obese women)	Transcriptomic analysis	Upregulation of 828 genes post-soda ingestion; inflammatory/metabolic gene activation.	[82]
Pregnant and lactating mice; rats	Gene expression (e.g., *PGP*)	Sucralose/acesulfame-K altered intestinal gene expression and transport activity.	[83,84]

**Table 3:** Natural sweeteners as potential alternatives to sucralose.

Sweetener	Natural Source	Relative Sweetness	Reference
Steviol glycosides	*Stevia rebaudiana*	~195 × sweeter than sucrose	[141]
Glycyrrhizin	*Glycyrrhiza glabra*	30–50×	[142]
Perillartine	*Perilla frutescens*	~2000×	[143]
Tagatose	Dairy-derived (e.g., lactose)	0.75 × (slightly less sweet)	[144]
Carob gum + tannin adducts	*Ceratonia siliqua*, *Prosopis glandulosa*	0.75×	[145]
Miraculin	*Synsepalum dulcificum*	Not sweet itself; modifies sour taste to sweet	[146]
Neohesperidin dihydrochalcone	Citrus-derived flavonoid	~3000×	[147]

## Abbreviations

The following abbreviations are used in this manuscript:

DMT1	Diabetes mellitus type 1
DMT2	Diabetes mellitus type 2
MODY	Maturity-Onset Diabetes of the Young
NSAID	Non-Steroid Anti-Inflammatory Drugs
